# Snow white

**DOI:** 10.1007/s12471-017-1020-1

**Published:** 2017-07-13

**Authors:** H. Lameijer, M. Kwant, M. Doff-Holman

**Affiliations:** Department of Emergency Medicine, University Medical Centre Groningen, University of Groningen, Groningen, The Netherlands

## Answer

The electrocardiogram of the question shows prolonged QRS and QTc segments. These symptoms can be seen in several types of intoxication, including poisoning by sodium-channel blockers such as tricyclic antidepressants and cocaine, but not in opioid intoxications. Therefore, a combined intoxication could be assumed. However, the most prominent abnormality in this electrocardiogram is the presence of J waves, best observed in the precordial leads. The J waves are known as Osborn waves, and are observed in hypothermia, hypercalcaemia, or Brugada syndrome [1, 2]. Osborn waves are thought to be caused by differences in action potential characteristics between the epicardial and endocardial layers of the heart [2].

Our patient’s core temperature was 31.8 degrees Celsius, which classifies as moderate hypothermia. Most serious complications, such as hypotension or ventricular fibrillation, occur below 28 degrees Celsius [3]. However, whenever electrocardiogram changes are observed, patients are at risk of life-threatening cardiac rhythm disturbances irrespective of the severity of hypothermia [2]. Therefore, caution is needed. Furthermore, patients should be rewarmed. Rewarming can be obtained by passive rewarming, such as hot blankets/air blankets, or active rewarming, such as warm intravenous fluids therapy, warm bladder or gastric rinses, or even warm extracorporeal membrane oxygenation.

**Fig. 1:**
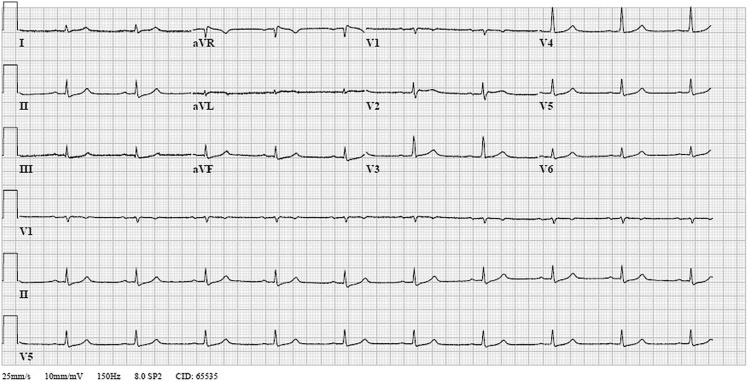
Electrocardiogram made during rewarming while the patient is still sedated and intubated. Minor J-point abnormalities are still present

**Fig. 2:**
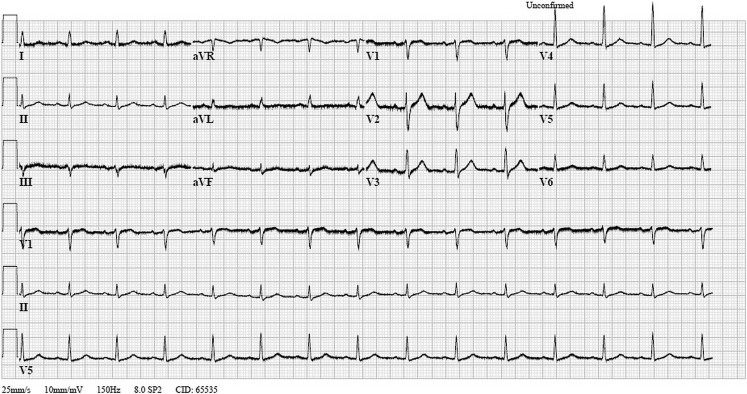
Electrocardiogram showing normalisation of J-point abnormalities. This electrocardiogram is made during rewarming after cessation of sedation and detubation, hence the shivering artefacts

Our patient was treated with warm intravenous fluids and hot air blankets. He was transmitted to the intensive care unit for further observation. A toxicology screening showed an opioid intoxication with methadone, no other intoxication was observed. After rewarming, his electrocardiogram normalised, as observed in Figs. 1 and 2.
